# Understanding Electrophoresis and Electroosmosis in Nanopore Sensing with the Help of the Nanopore Electro-Osmotic Trap

**DOI:** 10.1021/acsnano.4c04788

**Published:** 2024-07-25

**Authors:** Chenyu Wen, Sonja Schmid, Cees Dekker

**Affiliations:** † Department of Bionanoscience, Kavli Institute of Nanoscience, Delft University of Technology, Van der Maasweg 9, Delft 2629 HZ, The Netherlands; ‡ Laboratory of Biophysics, Wageningen University and Research, Stippeneng 4, Wageningen 6708 WE, The Netherlands

**Keywords:** nanopore, electro-osmosis, electrophoresis, capture rate, trapping potential, energy barrier, single-molecule technology

## Abstract

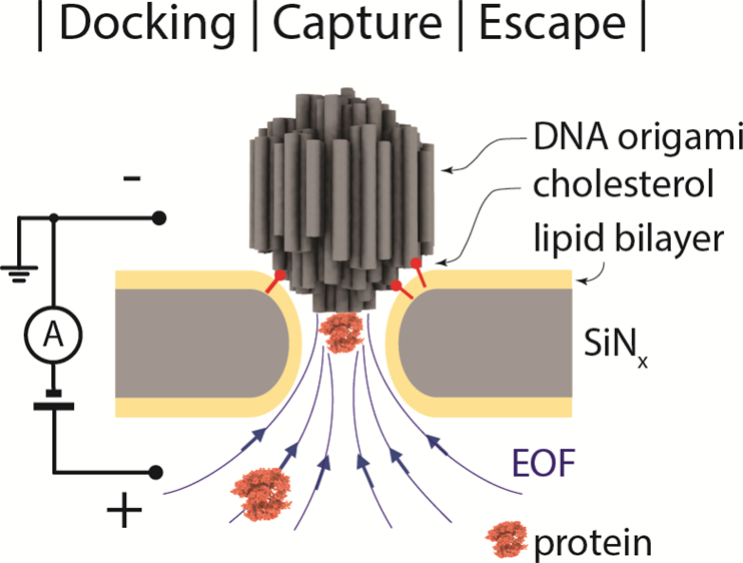

Nanopore technology is widely used for sequencing DNA, RNA, and peptides with single-molecule resolution, for fingerprinting single proteins, and for detecting metabolites. However, the molecular driving forces controlling the analyte capture, its residence time, and its escape have remained incompletely understood. The recently developed Nanopore Electro-Osmotic trap (NEOtrap) is well fit to study these basic physical processes in nanopore sensing, as it reveals previously missed events. Here, we use the NEOtrap to quantitate the electro-osmotic and electrophoretic forces that act on proteins inside the nanopore. We establish a physical model to describe the capture and escape processes, including the trapping energy potential. We verified the model with experimental data on CRISPR dCas9-RNA-DNA complexes, where we systematically screened crucial modeling parameters such as the size and net charge of the complex. Tuning the balance between electrophoretic and electro-osmotic forces in this way, we compare the trends in the kinetic parameters with our theoretical models. The result is a comprehensive picture of the major physical processes in nanopore trapping, which helps to guide the experiment design and signal interpretation in nanopore experiments.

## Introduction

**Figure 1:**
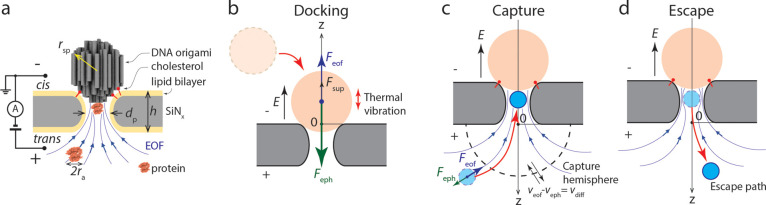
Configurations and definitions used in the NEOtrap model. (a) NEOtrap structure and working principle. (b-d) Schematics showing the processes of DNA-origami sphere docking, analyte capture, and a trapped analyte escape, respectively. The coordinate axis and origin are defined in each figure. To most conveniently express the physical quantities, positive direction is pointing upward in (b) and downward in (c) and (d).

Nanopore technology has been successfully applied to study various biomolecules at the single-molecule level, e.g., DNA/RNA sequencing,^1,2^ protein fingerprinting,^3,4^ peptide sequencing,^5,6^ and metabolite detection.^7,8^ The major kinetic processes, i.e., the capture, residence, and escape of analytes, universally exist in these nanopore sensing technologies. For example, in simple translocation experiments, target molecules are captured by the electric field whereupon they translocate through the nanopore;^9^ in some experiments with biological nanopores, proteins are captured and trapped by electro-osmotic flow (EOF);^10,11^ for DNA or peptide sequencing, a DNA helicase is docked onto a biological pore;^1,5^ in small solid-state nanopores, proteins are captured and escape again;^12^ for the detection of small chemicals, DNA-tethered streptavidin is docked on a nanopore;^7,13,14^ for the trapping of proteins, a DNA-origami structure is docked on solid-state nanopores,^15−17^ et cetera. This long list of examples illustrates the widespread impact and relevance of the coexisting kinetic processes in nanopore experiments, motivating a thorough review and a detailed model of these physical processes. The Nanopore Electro-Osmotic trap (NEOtrap) offers a perfect experimental platform to do this,^18^ since it involves all the aforementioned processes (Figure 1): docking of a DNA-origami sphere, capture of target proteins, and escape of the trapped proteins.

The NEOtrap^19^ is a label-free technology based on a simple setup illustrated in Figure 1a: a DNA-origami sphere is electrophoretically docked onto a passivated solid-state nanopore by an external bias voltage (Figure 1a) at the *Cis* side of the pore. This generates an EOF (upward in Figure 1a) since counterions at the surfaces of the DNA-origami sphere drag the surrounding water toward the negative pole. By utilizing this EOF, target proteins on the *Trans* side of the pore can be captured by the flow and trapped in the nanopore. The trapped protein partially blocks the through-pore ionic current which is detected as a change in the current level.^19^ Our previous work has shown that the NEOtrap can trap a wide variety of proteins with different molecular weights for a long time, even up to hours, and distinguish different conformations of them.^19,20^

Analytical models are critical to understand the driving forces and force balance in nanopore experiments, as they can reveal the underlying physics that causes experimental observations and thus provide a testable link between the theory and the experiment. Previous studies include simulations and analytic models for EOF generation in special nanopore shapes.^21^ Models were developed for the capture of particles by nanopores, considering electrophoresis, electro-osmosis, and dielectrophoresis.^22^ Thermodynamic models were developed to describe state transitions observed for α-hemolysin nanopores upon interaction with analytes such as β-cyclodextrin, poly(ethylene glycol), and short peptides.^23−25^

Here, we establish a framework of analytical models based on the NEOtrap that describes the three important processes in nanopore sensing in a generalized manner, viz., the capture of a target molecule (or nanostructure), its residence (or docking), and its escape. Furthermore, we present experimentally measured quantities of each process: the capture rates and release voltages of docked DNA-origami spheres, plus the capture rate and trapping times of CRISPR dCas9-RNA-DNA complexes, which serve here as a convenient platform to systematically modulate the charge and size of the analyte as described. These data satisfactorily verify the model. Our physical model applies to nanopore systems in general, reveals the interplay of the different phenomena, and facilitates the identification of critical parameters for experiments.

## Results and Discussion

### Electrophoretic Capture, Docking, and Induced Electro-Osmosis

We consider a DNA-origami sphere as a model analyte for electrophoretic capture in nanopores. The sphere carries a strong negative charge in an aqueous solution because of the deprotonated phosphate groups of the DNA backbone. Once the sphere enters the capture region of a nanopore, a strong electrophoretic force drives it to the nanopore and docks it onto a pore with a smaller diameter than the sphere. The capture of the origami sphere is a typical nanopore capture process, whose capture rate is proportional to the charge of the sphere and its concentration, as well as the electric field that is generated by the applied bias voltage (cf. details in the Supporting Information Note S1).

Once the sphere is docked on the nanopore, an upward EOF is generated. Due to thermal vibrations, the docked sphere is not statically positioned, but it vibrates constantly. In order to estimate the amplitude of this vibration, i.e., to evaluate the docking stability, we analyze the forces acting on a docked sphere. Three forces balance each other: the electrophoretic force holding the sphere on nanopore *F*_eph_, the shear force originating from the EOF *F*_eof_, and the support force from nanopore *F*_sup_. Considering the geometrical symmetry of the system, the force balance point should be right on the top of the nanopore, and we have
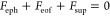
1

The electrophoretic force is
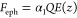
2where *Q* is the charge carried by the sphere which is negative here, α_1_ represents charge screening with a value between 0 and 1, and *E*(*z*) is the electric field intensity as a function of position *z*. In the coordinate system shown in Figure 1b, negative values represent the force pointing downward. *F*_eof_ can be expressed as the shear force from the water flow through the nanochannels inside the DNA-origami sphere as
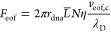
3where *r*_dna_ is the radius of the nanochannels, *L̅* the average length of the channels, *N* is the number of channels, η the viscosity of water, *v*_eof,c_ the (maximum) velocity of water molecules due to EOF inside the channels, and λ_D_ the Debye length. For geometrical reasons, *r*_dna_ can be approximated by the radius of double-stranded (ds) DNA, since the nanochannels result from the hexagonal honeycomb arrangement of the dsDNA strands in the DNA-origami sphere. *L̅* can be approximated by the diameter of the origami sphere 2*r*_sp_, and the EOF velocity is approximated to be linearly built up in the electric double layer (EDL) with a thickness of λ_D_. It is worth noting that the model is constructed for the vertical docking orientation of the DNA-origami sphere, which means that the DNA strands in the DNA-origami are perpendicular to the nanopore membrane. This was chosen because (i) the DNA-origami docking naturally shows a preference for the vertical orientation,^20^ which relates to the difference on the surface ion mobility in the moving direction parallel and perpendicular to the DNA stands;^26^ and (ii) cholesterol molecules were linked to precise positions on the surface of the DNA-origami sphere to lock the vertical docking orientation.

In our system, the nanopore is neutral in charge, since the silicon nitride is coated with a charge-neutral lipid bilayer to prevent nonspecific adsorption of analyte molecules. Thus, all of the EOF originates from the charge of the DNA-origami sphere. A simple way to estimate a model of the EOF velocity is provided by the formula for an infinitely long cylindrical tube with a uniform surface charge density^27^*v*_eof,c_ = ε_0_ε_r_ζ*E*(*z*)/η*,* where ε_0_ is the vacuum permeability, ε_r_ the relative permeability of water, and ζ the zeta potential of the channel wall. However, this estimation considers neither the size of the nanopore nor the nanochannels in the origami sphere. To be more precise, one should also consider the force balance acting on the water. It can be found that the support force given by the nanopore to the sphere *F*_sup_, equals the shear force given by the through-pore EOF to the nanopore wall (cf. the detailed explanation in Supporting Information Note S2), i.e.,
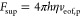
4where *h* is the thickness of the nanopore, and *v*_eof,p_ is the maximum velocity of the EOF in the pore located at the pore central axis. Because of the conservation of water flux, the volumetric rate through the pore should be equal to that through the nanochannels in the origami sphere, i.e.,
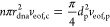
5where *d*_p_ is the nanopore diameter and *n* is the number of channels projecting to the pore region. From eqs 1 – 5, an expression of EOF velocity can be derived as a function of the electric field inside the nanopore *E*_0_ (the maximum of *E*(*z*)),
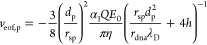
6
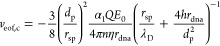
7

The forces are solved as well,
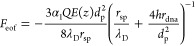
8
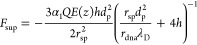
9

The detailed derivation can be found in the Note S3 in Supporting Information. Slip wall conditions commonly exist in nanopore/nanochannel systems,^28,29^ and are especially significant on surfaces that weakly interact with water molecules, e.g., a lipid bilayer.^30^ Therefore, a slip-wall effect can be considered in our model by introducing a slip length on the wall of the nanopore and the nanochannels in the origami sphere. Please see Supporting Information for the derivations and expressions considering this slip wall case (Note S4 in Supporting Information). We find that the EOF velocity can be significantly enhanced if the slip length is much larger than the dimensions of the nanopore.

To calculate the docking energy of a docked origami sphere, we need to integrate the driving force, i.e., *F*_eph_ + *F*_eof_, along a given escape path of the sphere, e.g., the central axis of the system as the simplest one. Since both forces, *F*_eph_ and *F*_eof_, are proportional to the electric field intensity *E*, the energy profile is defined if the distribution of *E* is known. The decay of the electric field from the mouth of the pore to infinity can be assumed to obey an inverse square relationship. (See Note S5 in Supporting Information).^31^ Thus, the docking energy profile can be obtained by the integration:
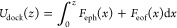
10

For integration, it is convenient to split eq 10 into two parts: within the pore (0< *z* < *d*_p_/2), we simply assume the electric field decreases linearly with distance z; outside the pore (*d*_p_/2< *z* < ∞), the equipotential surface can be approximated by a hemisphere, whose area increases quadratically with its:
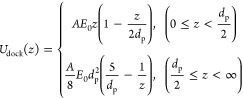
11with
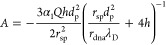
12

In thermal equilibrium, dispersed particles possess the same thermal energy as the surrounding medium molecules.^32^ Thus, the DNA-origami sphere carries a thermal energy of 1.5 *kT* as given by the equipartition theorem,^32^ where *k* is the Boltzmann constant and *T* is the temperature in Kelvin. The position distribution of a docked origami sphere due to thermal vibrations can thus be read from the energy profile by measuring the distance from the nanopore mouth to the position at which the energy reaches 1.5 *kT*. Furthermore, we can define a release voltage, *V*_res_, which is the minimum voltage required to hold the DNA-origami sphere in the docked state. This release voltage is defined as the voltage corresponding to an electric field causing a net docking potential (*U*_dock_) that is equal to the thermal energy experienced by the docked sphere (1.5 kT, i.e., the energy at infinite distance in eq 11: 5*AE*_0_*d*_p_/8 = 1.5 kT). From this, it follows that
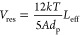
13where *L*_eff_ is the effective thickness of the nanopore.^33,34^

### Electro-Osmotic Capture

The upward-oriented EOF generates a shear force *F*_eof_ on analytes at the *trans* side of the nanopore, which can capture the analyte in the pore. If the analyte carries an electric charge, then an additional electrophoretic force *F*_eph_ acts on it. A capture hemisphere can be introduced (similar to earlier models of the capture process^19^), where the directional driving force and random Brownian motion balance each other. Once the analyte moves inside this capture hemisphere, the driving force dominates, leading to a higher probability for analyte capture than its diffusion away from the nanopore. The directional velocity of the analyte is
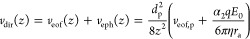
14where *q* is the charge carried by the analyte, α_2_ is the electrical screening factor for the analyte charge that has a value between 0 and 1, and *r*_a_ is the radius of the analyte. Here, *v*_eof,p_ can be calculated from the previous docking model (eq 7). The EOF decays in the same way as the electric field *E* from the nanopore to the infinite distance (i.e., with the same inverse square relationship, Note S5 in Supporting Information), since the electro-osmotic flux is also conserved on any iso-velocity hemisphere. The effective “diffusive velocity” is *v*_diff_ = 2*dD*/<*z*>, where *d* is the dimension of the system (*d* = 3 here) and <*z*> is the spatial range of diffusion in a time span *t*, which is proportional to the square root of the time span.^19^

The radius of the capture sphere R* is determined by the criterion that the “diffusive velocity” equals the directional velocity, which for *d* = 3 leads to
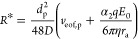
15

Thus, the capture rate can be expressed as^19^
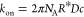
16

Substituting eqs 6 and 15 into eq 16 yields
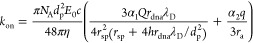
17

It is worth noting that a negative charge of the analyte (*q* < 0) induces an electrophoretic force in the opposite direction of the EOF, which retards the capture, i.e., yielding a reduced capture rate.

### Escape of a Trapped Analyte

We calculate the escape rate from the nanopore by considering that a trapped analyte can overcome an energy barrier through thermal vibrations with an average energy of 1.5kT. By integrating the net trapping force on a trapped analyte along its escape path, we can calculate the energy profile of the trapping potential (cf. docking energy profile in Section 2.1). The driving force on a charged analyte is the superposition of electrophoretic force, *F*_eph_, and electro-osmotic force, *F*_eof_, which can be expressed as
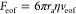
18
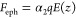
19

Substituting eq 6 into eq 18, the driving force can be expressed as
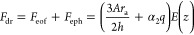
20

Thus, the trapping energy profile is^19^
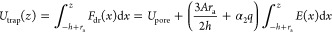
21

Inside the pore (*z* < 0), the driving force is assumed to be constant at its maximum, and the trap depth is *h* – *r*_a_. Thus,
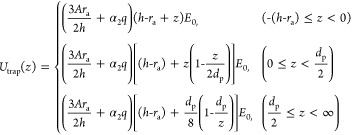
22

The height of the trap’s energy barrier is defined as the energy at the position of the capture radius, i.e., *z* = R*,
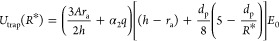
23

This yields the trapping time constant τ_trap_ as the reciprocal of the escape rate, *k*_off_, which follows the Arrhenius relationship.
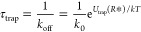
24

### Validation: Electrophoretic Capture

**Figure 2:**
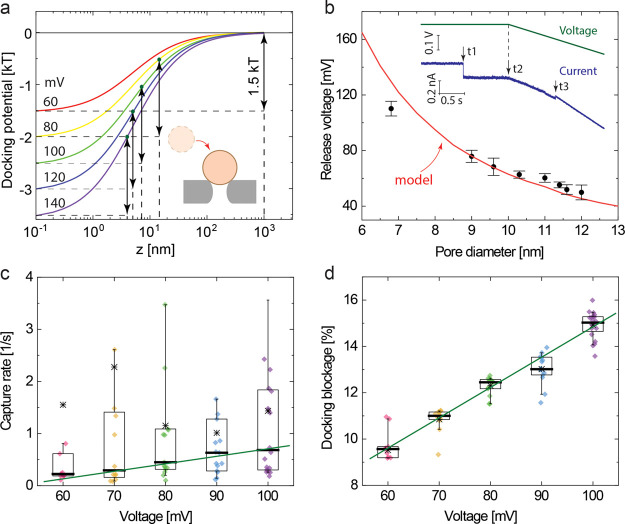
Docking of the DNA-origami sphere. (a) Docking potential of a 35 nm diameter sphere onto a 10 nm diameter pore at different voltages according to eq 11. The zero potential is set at an infinite distance. Arrows indicate the thermal vibration energy in 3D (1.5kT) with respect to the potential minimum, and the vertical dashed lines link it to the vibration amplitude, indicating by the dots, of the docked sphere in the *z*-direction. (b) Experimental release voltage of a docked origami sphere on a nanopore with different sizes. Dots with error bars denote measured values, showing the means and standard deviations of 20–30 escape events under each condition. The solid line shows the fitting results from our model (eq 13) with one fit parameter, the charge screening factor of DNA, α_1_. Inset: typical example traces of ionic current and applied voltage in release-voltage measurements. A sphere is docked at *t*_1_ by a 200 mV voltage, at *t*_2_, a negative voltage ramp starts, and at *t*_3_, the sphere is released. (c, d) Capture rate of the origami spheres and relative current blockage caused by the docking at different voltages, respectively. The dots represent individual docking events, and the box chart shows their mean (asterisk), median (thick bar), and 10, 25, 75, and 90% percentiles. Solid lines are linear fits to the median values.

We studied the docking process of the DNA-origami sphere at various voltages in SiN_*x*_ nanopores of different sizes. In these measurements, we used bare DNA-origami spheres without cholesterol anchors. Figure 2a shows the energy potential of docking according to our model for varied applied voltages and a 10 nm diameter pore (other parameters can be found in Table S1 in Supporting Information). The zero potential is set at an infinite distance. With increasing voltage, the force that holds the docked origami sphere on the nanopore also increases, causing a deeper potential well and thus a higher energy barrier for escape. Considering the docking energy at 1.5 kT (i.e., the energy of spatial vibrations in 3D), yields an estimate of the spatial amplitude of the sphere’s thermal vibrations. Specifically, we find vibrational amplitudes of 4, 5, 7, and 13 nm at 140, 120, 100, and 80 mV, respectively. For 60 mV bias, the docking potential barely reaches 1.5 kT, indicating that at 60 mV, the sphere is not stably docked onto the nanopore, thus defining the release voltage.

This theoretical description is supported by experimental measurements of the release voltage, where we first docked the sphere using a large voltage (e.g., 200 mV here for a 6.8 nm pore) and then gradually ramped down the voltage. In the example provided in the inset of Figure 2b, a downward current step occurs at *t*_1_, indicating sphere docking; at *t*_2_, the voltage ramp starts, followed by a current increase at *t*_3_, indicating the escape of the sphere from the pore. The voltage at *t*_3_ is the release voltage, as displayed for different pore sizes in Figure 2b. The modeling results from eqs 12 and 13 (solid line in Figure 2b) predict the experimental observation very well with only one free fit parameter, viz., the charge screening factor α_1_ which we found to be α_1_ = 0.3 ± 0.084. This value is comparable to previous literature reporting for a linear DNA duplex (0.25 ± 0.013).^35^ Since the size of counterions, K^+^ here, is much smaller than the interval space among DNA duplexes in the origami, the origami folding likely does not change this screening factor significantly. During the fitting, many parameters were given by the nanopore/DNA-origami used (e.g., *Q*, *h*, *r*_sp_, and *r*_DNA_) or by the experimental conditions (*c* and λ_D_). These were kept constant, and their numbers can be found in Table S1 in Supporting Information. The trend of the decreasing release voltage with increasing pore diameter can be understood from the fact that the origami sphere fits deeper into the larger pore where the electric field is stronger, and correspondingly, the holding force is stronger and the release voltage is smaller.

Furthermore, we measured the capture rate of the origami sphere at different voltages, as shown in Figure 2c. Each dotted line in the figure represents an individual docking event. The capture rate is the reciprocal of the waiting time between voltage application and observed docking. The medians of the distributions show a clear linear dependence on voltage, which agrees well with the theoretical prediction that the capture rate is proportional to the electrical field intensity (see Supporting Information Note S1).

The current blockade (i.e., the percentage of the current drop compared to the open-pore current) caused by the docking also shows a linear dependence on the voltage (Figure 2d). This trend can be explained by the fact that a higher voltage generates a stronger force, pushing the docked sphere deeper into the nanopore, thereby deforming it and causing a larger current blockade. The blockade shows ∼10% variation that could be induced by the unlocked docking orientation during the experiment, although the DNA-origami sphere possesses a preference of the vertical docking orientation.

### Validation: Electro-Osmosis Dominated Capture

**Figure 3:**
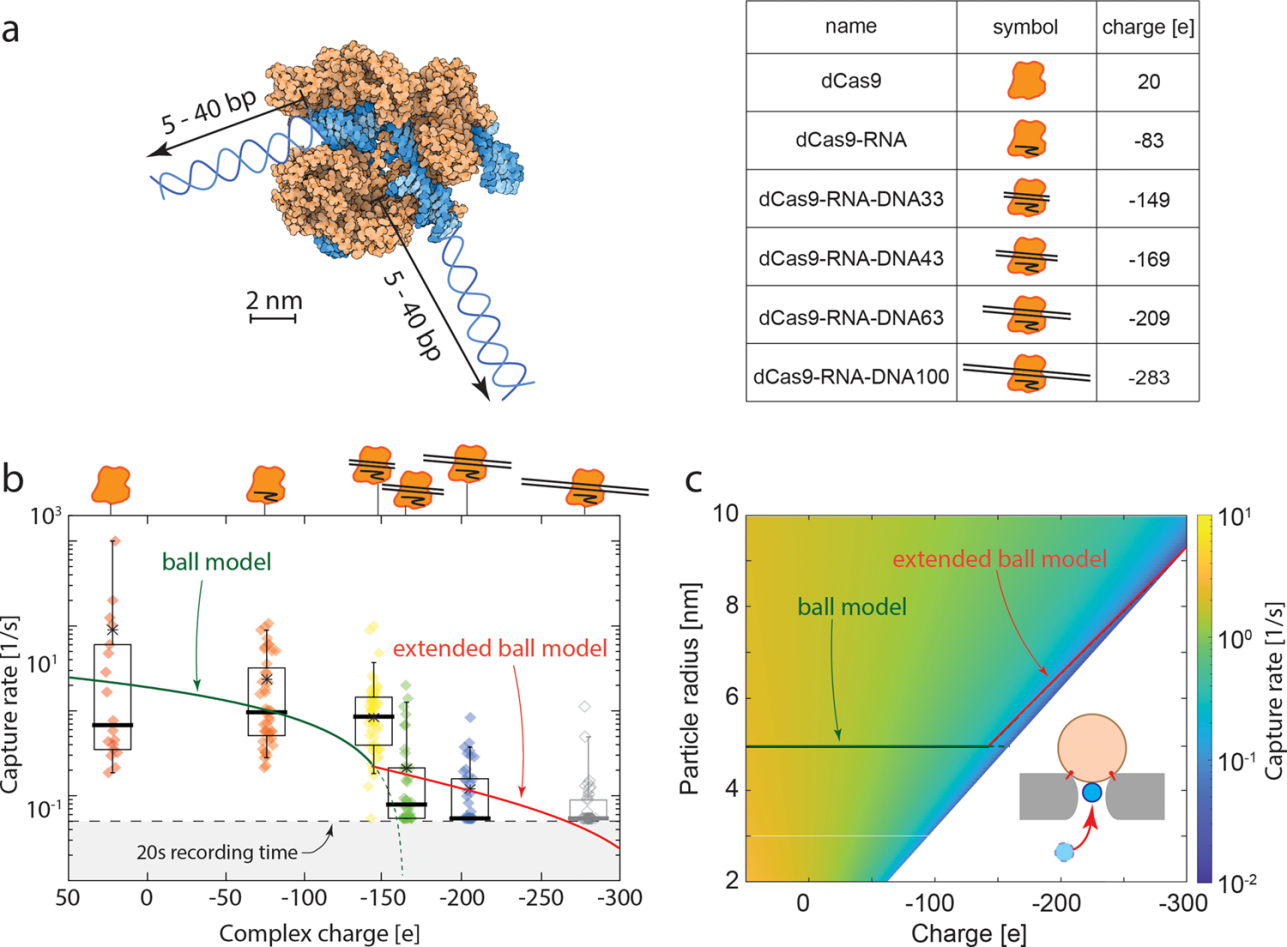
Nanopore capture of the protein dCas9 and its complexes. (a) Left: 3D structure of the dCas9-RNA-DNA complex showing the protein (orange) and nucleic acids RNA and DNA (blue) (PDBID: 6O0X); right: a table listing the six samples used in our trapping experiment with their names, symbols, and charge numbers. (b) Capture rate of dCas9 and its complexes measured in the NEOtrap for a 15 nm diameter nanopore at 100 mV voltage. The concentration of each sample is 5 nM. Each dot represents an individual capture event, and the box chart displays the mean (asterisk), median (thick bar), and the 10, 25, 75, and 90% percentiles. The gray shaded area is beyond the experimental limit set by the 20 s recording time. Solid curves show the modeling results. The green segment represents a fit of the *ball model*: eq 17, with sample size fixed to 10 nm diameter, two free parameters, and the dashed tail extrapolates beyond the experiment condition. The red segment represents the *extended model*: eq 17, with simultaneously increasing size and negative charge of the analyte. (c) Predicted capture rates for analytes with varied charges and radii. Solid lines show the fitting results in (b) with the same color code. In the white area, the (opposing) electrophoretic force dominates, and thus the proteins cannot be captured.

To verify our capture model, we performed an extensive experimental study of various dCas9-DNA complexes with the NEOtrap system, as illustrated in Figure 3a. Dead Cas9 (dCas9, i.e., DNA cleavage inactivated) was measured alone, preincubated with its guide RNA only, or also with its target dsDNA of various lengths. By systematic variation of the length of the dsDNA, we tune the size and the charge carried by the complex. Six different complexes were prepared, viz., pure dCas9, dCas9-RNA, and dCas9-RNA-DNA with 33 base pairs (bp), 43, 63, and 100 bp, and used for trapping experiments. The respective charges carried by dCas9 and those complexes are listed in the table in Figure 2a (see Note S6 in Supporting Information for details). Details about the sample preparation can be found in the Method section. The dCas9 complexes were trapped in a 15 nm diameter nanopore at a 100 mV bias voltage. In this measurement, we used the cholesterol-functionalized DNA-origami sphere to control a vertical docking orientation.^20^ Trappings were reversibly repeated 20 times in each condition. Twenty-second current traces were recorded in each round of the trapping.

The results for the capture rates of these analytes are shown in Figure 3b as a function of their charge. The solid line shows the fitting results from our model (eq 17). In general, the model follows the trend observed in the experiment and thus grasps the major physics of the capture process. The kink in the curve at −143e charge divides the curve into two segments with different charge dependences dictated by the experiment. Before this point, the length of bound dsDNA in the dCas9 complex is shorter than the size of dCas9 (2*r*_a_ = 10 nm), and thus the overall size of the complex is the same as the dCas9 size, i.e., a 10 nm diameter ball. In this range, the ball model is applied. However, after this kink, the dsDNA is longer than the size of dCas9, and therefore, the dsDNA will protrude from the protein. Here, the size and charge of the complex change simultaneously, and the extended ball model is applied in this region. To avoid complicating our model and involving too many free parameters in the extended ball model, we also approximated this complex with a sphere, however, with an effective radius that is larger than in the ball model. While details of the morphology and orientation of a trapped complex are ignored by this approximation, the extended ball model does capture the experimentally observed capture rates beyond −150 e, i.e., beyond the applicable range of the ball model. This slower decrease of the capture rate with increasing negative charge is expected since the growth in size (dsDNA protrusions) causes an increase of *F*_eof_ which partially compensates for the increasing retardation of *F*_ehp_. We adopted the previously found DNA charge screening factor α_1_ = 0.3 and fitted two parameters: the charge screening factor of the complexes, α_2_ = 0.25 ± 0.1, and the scaling factor of the effective radius of the protein-dsDNA complex with protruding dsDNA, *f*_eff_ = 0.27 ± 0.23 (cf. Note S7 in Supporting Information). The rest of the parameters were set by the materials and measurement conditions and kept constant during fitting (cf. Table S1 in Supporting Information). We note that the measurement of the capture rate of dCas9-RNA-DNA100 was limited by the 20 s recording time and was therefore omitted from the fit.

Our model (eq 17) can describe the capture rate of various spherical or near-spherical proteins and protein complexes with different charges and sizes, as visualized in Figure 3c. The parameters used in the calculation are listed in Table S1 in Supporting Information. In general, we find higher capture rates for larger protein sizes with smaller negative charges. The larger size causes a stronger *F*_eof_, while the smaller negative charge induces less retardation by *F*_eph_ (opposing *F*_eof_). The dependences of capture rate on bias voltage, analyte concentration, and nanopore size were also evaluated in Ovalbumin trapping experiments. As predicted from our model, the capture rate was found to be linearly dependent on the bias voltage and analyte concentration (cf. eq 17), as shown in Figures S4a and S5 in Supporting Information. These results are intuitive since a higher voltage generates a higher electric field which indicates a stronger EOF as well as a stronger driving force. Moreover, the higher analyte concentration induces a higher frequency of analytes that enter the capture region. The dependence of the capture rate on the nanopore size can be complicated (cf. eq 17). However, in general, the capture rate shows a monotonic positive correlation with nanopore size, i.e., the larger the size of the pore, the higher the capture rate (Figure S6a,c in Supporting Information). This can be understood from the fact that more nanochannels of the DNA-origami sphere project to a larger nanopore, thus increasing the EOF through the pore, yielding a larger driving force on the analyte.

### Validation: Diffusion-Driven Escape

**Figure 4:**
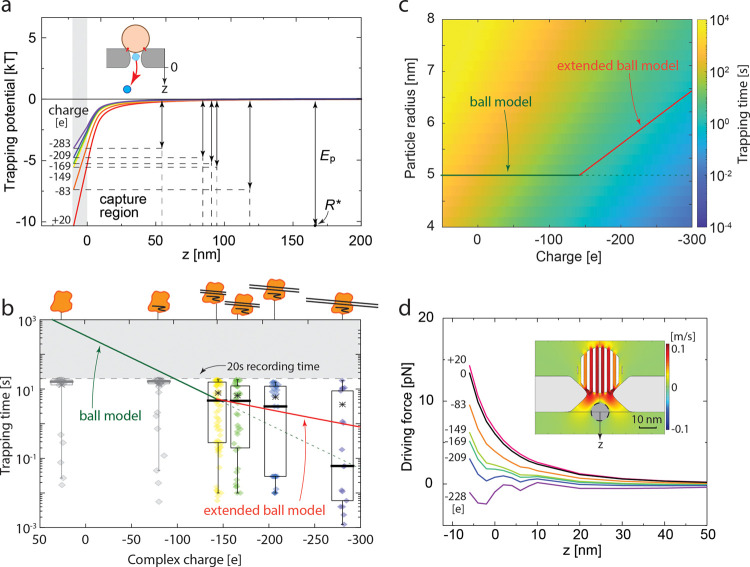
Nanopore escape of the trapped protein dCas9 and its complexes. (a) Trapping potential as a function of z distance for a 10 nm diameter sphere of varied charge, trapped in a 15 nm diameter pore at 100 mV. Zero potential is set at the infinite distance. The gray shaded area marks the pore region (cf. coordinate definition in the inset). Vertical dashed lines indicate the capture radius R* for each condition, and the arrows mark the corresponding trapping energy, *U*_p_, with respect to the energy minimum (horizontal dashed lines). The capture region is the zone within the capture radius in which the directional driving force governs the protein movement. (b) Trapping time of dCas9 and its complexes measured in the NEOtrap for a 15 nm diameter nanopore at 100 mV. The concentration of each sample is 5 nM. Each dot represents an individual trapping event, and the box chart shows the mean (asterisk), median (thick bar), and 10, 25, 75, and 90% percentiles. The gray shaded area is beyond the experimental limit set by the 20 s recording time. The green segment represents a fit of the *ball model*: eqs 22–24, with sample size fixed to 10 nm diameter, two free parameters, the dashed tail extrapolates beyond the experiment condition. The red segment represents the *extended model*: eqs 22–24, with simultaneously increasing the size and negative charge of the analyte. (c) Predicted trapping times for analytes of varied charge and radius. The solid lines show the fitting results in (b) with the same color code. (d) COMSOL simulation results of the driving force on a 10 nm diameter sphere with different charges along the central axis of the NEOtrap system with a 15 nm diameter nanopore at a 100 mV bias voltage. Inset: EOF velocity distribution in the NEOtrap system with a 15 nm diameter nanopore and a 10 nm diameter sphere at the mouth of the nanopore (*z* = 0 position). Warm color indicates the upward velocity, while the cold color indicates the downward velocity (cf. color scale).

Our model also describes the trapping potential and escape energy barrier of a trapped analyte; see eq 22. As visualized in Figure 4a, with a decrease of the negative charge of the analyte, the potential energy becomes deeper and the capture hemisphere becomes larger. The zero potential is set at the infinite distance. The higher trapping energy, *U*_p_, implies a longer trapping time. In the pore region (gray shadow region in Figure 4a), the energy increases linearly with distance, as we assume a constant maximum driving force inside the pore. Furthermore, the energy increases rapidly form the potential well in the capture region while leveling out in the free diffusion region outside of the capture region (cf. eq 22).

From the same trapping experiments described in Figure 2, we can extract the trapping time; see Figure 4b. We observe a lower trapping time for increasingly larger negative charges. The solid line is a fit of eq 24, with a kink at the −143e position where the ball model and extended ball models meet (cf. Figure 3b). In the model, we adopted the previously found charge screening factors of DNA and protein α_1_ = 0.3 and α_2_ = 0.25, and fitted parameter the scaling factor of the dependence of the effective radius of the complex on DNA length *f*_eff_ = 0.12 ± 0.1, and the rate constant *k*_0_ = 45 ± 22/s in the Arrhenius relationship (eq 24). For *f*_eff_, we did not inherit the value from the capture model because in the capture process, the complex can freely tune its angle, while in the escape mode here, the trapped complex is basically vertically stuck in the nanopore. Thus, in these two models, the effective size of the complex can be different. The remaining parameters were kept constant during fitting (cf. Table S1 in the Supporting Information). The model reproduces the experimental trend despite appreciable scattering of the exponentially distributed experimental data. Since the trapping times of the least charged dCas9 and dCas9-RNA complex were frequently longer than the experimental recording time (20 s), these data points were omitted from the fit (gray data in Figure 4b).

Figure 4c shows the trapping time as predicted by our model (eq 24) for spherical proteins/protein complexes with varied charges and radii. A lower negative charge and larger protein radius result in a stronger driving force and longer trapping time (cf. analysis for Figure 3b). For comparison, the trapping time of Ovalbumin was also measured at different voltages and with various pore sizes. As predicted from our model, the trapping time depends exponentially on the bias voltage (cf. eqs 23 and 24), as shown in Figure S4b in Supporting Information. Furthermore, a larger pore caused a longer trapping time (Figure S6b,d in Supporting Information), again in agreement with our model (cf. eqs 23 and 24).

Finally, we numerically extracted the forces acting on a particle in the NEOtrap system using COMSOL simulations. The detailed configurations of the simulation can be found in the Methods section, and all parameters are listed in Table S1 in the Supporting Information. Figure 4d shows the total driving force on a 10 nm diameter protein during its escape from the nanopore. The trapping force appears to be of the order of a few pN. The inset shows the distribution of EOF velocity generated by a DNA-origami sphere docked at a 100 mV bias voltage. As expected, the force rapidly decreases when the particle moves away from the pore. For a smaller negative charge of the particle, a stronger driving force is obtained. The COMSOL results support our understanding of the physics, as discussed above.

## Conclusions

In our work, we established three physical models to describe electrophoretic capture, electro-osmosis dominated capture, and diffusion-driven escape by considering the interplay among diffusion, electrophoresis, and electro-osmosis in the system. The models offer fair predictions for the docking energy, the capture radius, and the trapping energy, which we could verify with measurable quantities, viz. the release voltage of a docked sphere, the capture rates of a variety of dCas9 complexes, and the trapping times of these complexes, as obtained from a set of experiments.

The frameworks of capture, docking, residence, and escape are universally relevant in nanopore sensing systems and can be easily transferred to other systems by adapting the expressions of the respective driving forces. For example, almost all nanopore systems entail a capture process and can involve different driving forces besides EOF and electrophoresis,^36^ e.g., diffusioosmosis,^37^ diffusiophoresis,^38^ or thermal gradients.^39^ Upon substitution of expressions for these driving forces in eq 14, the model generalizes to describe the corresponding capture rate. In addition, the escape model can also be used to describe single-molecule trapping in other systems, e.g., protein pores, such as ClyA,^10^ YaxAB,^40^ MspA,^11^ or also bare solid-state SiN_*x*_ nanopores,^12^ by substituting the expressions for the driving force in eq 21. Finally, the docking mode can be used to evaluate the docking stability of other objects in a nanopore, such as a DNA helicase in MspA for DNA/peptide sequencing,^1,5^ a protein–DNA/-peptide structure in a nanopore for nucleotide/amino acid analysis,^11,14,41^ a biological pore on a solid-state pore,^17^ and a DNA-origami structure on a solid-state pore.^15^

Overall, this work elucidates electrophoretic capture, electro-osmosis-dominated capture, as well as diffusion-driven escape in general nanopore systems. It provides a theoretical model with experimentally testable quantities that were verified herein. Altogether, using the NEOtrap as a convenient test system, we present a theoretical foundation aimed to guide the experimental design and signal interpretation of single-molecule nanopore experiments of all kinds.

## Methods

We also refer to the Methods section of refs (20) and (19). Preparation of the DNA-origami sphere can be found in ref (20).

### Nanopore and Ionic Current Measurement

The nanopores are drilled by a transmission electron microscope (Titan aberration-corrected TEM, Thermo Fisher Scientific, USA) in freestanding 20 nm-thick SiN_*x*_ membranes deposited on glass chips.^42^ Then, the nanopore devices were rinsed with deionized water (DIW, Milli-Q, Merck KGaA, Germany), ethanol, acetone, isopropyl alcohol, and DIW, in sequence as mentioned. All chemicals were purchased from Merck unless stated otherwise. Afterward, they were further cleaned by plasma (SPI Supplies Plasma Prep III, USA) before being mounted in a custom-made polyether ether ketone (PEEK) sample holder with an electrolyte reservoir at each side of the nanopore and corresponding fluidic tubing. The entire setup is placed in a Faraday cage for screening of electromagnetic interference during the electrical measurement. The electrolyte in the two reservoirs is electrically connected to an Axopatch 200B amplifier (Molecular Devices LLC, UK) using Ag/AgCl electrodes (silver wire chloridized in household bleach). The analog signals are digitalized by a Digidata 1550B digitizer (Molecular Devices LLC, UK) and recorded by a computer with software Clampex 10.5 (Molecular Devices LLC, UK). After both chambers were flushed with DIW, the chambers were filled with 600 KHM buffer (600 mM KCl, 50 mM Hepes, 5 mM MgCl_2_, pH 7.5) for current–voltage (*I–V*) measurements (voltages ranging from −120 to 120 mV). The diameter of the nanopores was extracted from their conductance using the simple model described in.^19,33^All measurements were performed under 500 kHz sampling and a 100 kHz low-pass filter (four-pole internal Bessel filter) at a room temperature of 21 ± 1 °C.

### Lipid Bilayer Coating

In order to prevent the nonspecific adsorption, surface passivation of the pore was implemented by using 1-palmitoyl-2-oleoyl-*sn*-glycero-3-phosphocholine (POPC, Avanti Polar Lipids Inc., USA). Vials with POPC in chloroform were dried in a vacuum and subsequently stored at −20 °C. Before usage, the lipids were resuspended in 600 KHM buffer (600 mM KCl, 50 mM Hepes, 5 mM MgCl_2_, pH 7.5) to a concentration of 1 mg/mL. The suspensions were then sonicated with a pin sonicator (Qsonica, USA) for 15 min (33% duty cycle, 20% power). Then, 50 μL of lipid suspension was added to the ground-side reservoir of the nanopore while applying an AC voltage with a triangle waveform, a 50 mV peak amplitude, and a 1 Hz frequency. Coating with a lipid bilayer will cause an increase of resistance, which is reflected in the decrease of the amplitude of the corresponding current. After incubation for 10 min, the entire sample holder was totally immersed in DIW. Under water, the chambers were flushed with DIW and incubated for 20 min. Then, the chambers were flushed with DIW again before the holder was taken out of the bath, dried externally, filled with 600 KHM, and reconnected to the amplifier. Finally, the *I*–*V* curve was measured again, and in comparison with the *I–V* before coating, the size of the nanopore before and after coating can be extracted.

### Protein Sample Preparation

dCas9 proteins were purchased from Integrated DNA Technologies, Inc. (Alt-R S.p. dCas9 Protein V3,1081066, USA). All of the RNA and DNA were purchased from Ella Biotech GmbH (Germany). The sequences of all the nucleic acid samples are listed below. First, crRNA and tracrRNA were mixed in the duplex buffer (100 mM potassium acetate, 30 mM HEPES, pH 7.5) with a concentration of 10 μM and gradually cooled (from 95 to 25 °C, 5 °C step, 5 min stay in each step) to form the Tr-crRNA hybridized structure. Second, the 550 nM dCas9 was mixed with 2 μM Tr-crRNA in NMH buffer (100 mM NaCl, 50 mM Hepes, 10 mM MgCl_2_, pH 7.5) and incubated at 37 °C for 10 min to form the dCas9-RNA complex. Third, the 50 nM dCas9-RNA complex was mixed with 500 nM DNA (5 different lengths in separate reaction tubes) in NHM buffer and incubated at 37 °C for 30 min to form the final dCas9-RNA-DNA complex. The dCas9, dCas9-RNA, and dCas9-RNA-DNA samples were future dispersed in 600 KHM buffer with a 5 nM concentration for the NEOtrap measurements.

Protein Ovalbumin from the Gel Filtration Calibration Kits was purchased from Cytiva LLC. (USA) and dispersed in 600 KHM buffer with a target concentration.

crRNA: 5′-GGCAUCGGUCGAGGAACUUUCGG-3′ + 13 nt fixed sequence from the company = 36 nt

tracrRNA: 67 nt fixed sequence from the company.

DNA33: 5′-GCGGAGGCATCGGTCGAGGAACTTTCGGGTGTG-3′

DNA43: 5′-CACACAGGGAGGCATCGGTCGAGGAACTTTCGGGTGTAGAAAC-3′

DNA63: 5′-TGGTGACACTCACACAGGGAGGCATCGGTCGAGGAACTTTCGGGTGTAGAAACTGCCG GAAAT-3′

DNA100: 5′-GGACACGCCTAAATCAACGCTGGTGACACTCACACAGGGAGGCATCGGTCGAGGAACTTTCGGGTGTAGAAACTGCCGGAAATCGTCGTGGTATTCACTC-3′

### Data Processing

The data was analyzed using self-written MATLAB code. For the detection of trapping events, the function ‘*findchangepts*’ was adopted to find the time points of the current level changes between the docking state and trapping state. Information about each trapping event, including the duration, blockage amplitude, and interval between the two adjacent events, was extracted. In addition, an amplitude threshold for the event detection was placed at three times the standard deviation of the noise of the current baseline with docked DNA-origami.

### COMSOL Simulation

Numerical simulations of the NEOtrap system are implemented on COMSOL Multiphysics 5.4 with a two-dimensional axial symmetrical domain. The simulation includes the fluid domain, the membrane domain, the DNA-origami sphere domain, and the analyte domain, whose relative permittivity was set to 80, 7.5,^43^ 8.3,^44^ and 3.2^45^ for water, SiN_*x*_, DNA, and protein analyte, respectively. The ion distribution and movement in an electrolyte was governed by the Nernst–Planck equation, the electric potential distribution was described by the Poisson equation, and the fluid flow was determined by the Navier–Stokes equations. The *Transport of Diluted Species* module (Nernst–Planck equation), the *Electrostatics* module (Poisson equation), and the *Laminar Flow* module (Navier–Stokes equations) were incorporated and fully coupled in the simulation. The electrolyte is 600 mM KCl with the mobilities of K^+^ and Cl^–^ being 7.0 × 10^–8^ and 7.2 × 10^–8^ m^2^ V^–1^ s^–1^, respectively.^46^ The respective diffusion coefficient was then determined through the Einstein relation. During the simulation, the analyte was placed at different positions along the central axis, and the electrophoretic force and electro-osmotic force are calculated by integration of the electric surface stress tensor, *es.unTe*, and the total stress tensor, *spf.T_stress*, on the analyte surface.

## Supporting Information Available

Capture of DNA-origami sphere; force balance and EOF calculation of a docked DNA-origami sphere; consideration of the slip wall condition; distribution of electric field; charge of the dCas9-RNA-DNA complexes; effective radius of the dCas9-RNA-DNA complexes; parameters of models and their values used in the fitting; trapping protein Ovalbumin at various voltages; capture of Ovalbumin with different concentrations; and trapping Ovalbumin by nanopores with different sizes (PDF)
