# Supplementary material for: Understanding Electrophoresis and Electroosmosis in Nanopore Sensing with the Help of the Nanopore Electro-Osmotic Trap
Source: ACS Nano. 2024 Jul 25;18(31):20449–58. doi: 10.1021/acsnano.4c04788 (PMC11308919; doi:10.1021/acsnano.4c04788)
Supplement: Supplementary file 1 — nn4c04788_si_001.pdf [file nn4c04788_si_001.pdf]

**Supporting Information**

# Understanding Electrophoresis and Electroosmosis in Nanopore Sensing with the Help of the Nanopore Electro-Osmotic trap

Chenyu Wen[1], Sonja Schmid[2*], Cees Dekker[3*]

1. Department of Bionanoscience, Kavli Institute of Nanoscience, Delft University of Technology, Van der Maasweg 9, Delft, 2629 HZ, The Netherlands; Laboratory of Biophysics, Wageningen University & Research, Stippeneng 4, Wageningen, 6708 WE, The Netherlands; Present address: Division of Solid-State Electronics, Department of Electrical Engineering, Ångströmlaboratoriet, Uppsala University, Lägerhyddsvägen 1, 75237 Uppsala, Sweden.
2. Laboratory of Biophysics, Wageningen University & Research, Stippeneng 4, Wageningen, 6708 WE, The Netherlands; Present address: Department of Chemistry, University of Basel, Mattenstrasse 22, 4058 Basel, Switzerland.
3. Department of Bionanoscience, Kavli Institute of Nanoscience, Delft University of Technology, Van der Maasweg 9, Delft, 2629 HZ, The Netherlands.

*Corresponding authors: c.dekker@tudelft.nl, sonja.schmid@unibas.ch

**Table of contents**

Figure S1. Schematics showing the force balance of electroosmotic systems.

Figure S2. Schematics showing the volume of a docked DNA origami sphere.

Figure S3. Schematics showing the velocity profiles in a tube.

Figure S4. Trapping the protein Ovalbumin at various voltages.

Figure S5. Trapping Ovalbumin with different concentrations.

Figure S6. Trapping Ovalbumin by nanopores with different sizes.

Table S1. Fitting parameters and their values.

Note S1. Capture of the DNA origami sphere.

Note S2: Force balance for a docked DNA origami sphere.

Note S3: Electroosmotic flow calculation in the NEOtrap system

Note S4: Consideration of the slip wall condition.

Note S5: Distribution of the electric field.

Note S6: Charge of various dCas9-RNA-DNA complexes.

Note S7: Effective radius of the dCas9-RNA-DNA complexes.

**Supporting Table S1:** Model parameters and their values used also for analytical model fitting

| **Name** | **Symbol** | **Value** | **Unit** |
|---|---|---|---|
| Boltzmann constant | $k$ | $1.38\times10^{-23}$ | [J/K] |
| Temperature | $T$ | 300 | [K] |
| Element charge | $e$ | $1.6\times10^{-19}$ | [C] |
| Avogadro constant | $N_A$ | $6.02\times10^{23}$ | [/mol] |
| Permittivity of vacuum | $\varepsilon_0$ | $8.85\times10^{-12}$ | [F/m] |
| Relative permittivity of water | $\varepsilon_r$ | 80 | 1 |
| Viscosity of water | $\eta$ | $10^{-3}$ | [Pa·s] |
| Salt concentration of electrolyte | $c_0$ | 600 | [mM] |
| Analyte concentration | $c$ | 5 | [nM] |
| Charge of origami sphere[1,2] | $Q$ | 15120 | [e] |
| Screening factor of the charge on DNA[3] | $\alpha_1$ | 0.3 | 1 |
| Charge of protein and complexes | $q$ | -283 - +20 | [e] |
| Screening factor of the charge on protein | $\alpha_2$ | 0.25 | 1 |
| Radius of DNA origami sphere[1,2] | $r_{sp}$ | 17.5 | [nm] |
| Total number of nanochannels in the origami[1] | $N$ | 37 | 1 |
| Radius of the nanochannels | $r_{dna}$ | 1.1 | [nm] |
| Radius of protein | $r_a$ | 5 | [nm] |
| Diameter of nanopore | $d_p$ | 15 | [nm] |
| Effective thickness of nanopore | $h$ | 15.6 | [nm] |
| Applied bias voltage | $V$ | 0.1 | [V] |
| Increase rate of the effective radius of protein-dsDNA complex with prolong dsDNA | $f_{eff}$ | 0.12-0.27 | 1 |
| Rate constant | $k_0$ | 45 | [1/s] |

**Supporting Note S1: The capture of DNA origami sphere**

The capture process of the analyte is a competition between a directional driving force and random Brownian motion of the analyte, as described in the main text. Considering the spatial range <z> of a randomly walking particle in a time span *t*, an effective "diffusive velocity" can be defined as:

$$v_{diff} = \frac{\langle z \rangle}{t} \tag{S1}$$

According to the Brownian motion model, we have

$$\langle z^2 \rangle = 2dDt \tag{S2}$$

$$v_{diff} = \frac{2dD}{z} \tag{S3}$$

where, *d* is the dimension of the system, and *D* the diffusivity of the particle, *z* the displacement of the particle. For the capture of a DNA origami sphere, the driving force is simply the electrophoretic force:

$$F_{eph} = \alpha_1 QE \tag{S4}$$

According to Stokes' law, the electrophoretic velocity can be written as:

$$v_{eph} = \frac{F_{eph}}{6\pi\eta r_{sp}} = \frac{\alpha_1 QE}{6\pi\eta r_{sp}} \tag{S5}$$

As discussed in the main text, the decay of *E* from the nanopore center to the infinite distance follows the inverse square rule. Thus:

$$v_{eph}(z) = \frac{\alpha_1 QE_0}{6\pi\eta r_{sp}} \frac{d_p^2}{8z^2} \tag{S6}$$

On the capture hemisphere, the electrophoretic velocity is equal to the "diffusive velocity", and the radius of the capture hemisphere can be derived.

$$\frac{2dD}{R^*} = \frac{\alpha_1 QE_0}{6\pi\eta r_{sp}} \frac{d_p^2}{8R_{sp}^{*2}} \tag{S7}$$

$$R_{sp}^* = \frac{\alpha_1 QE_0}{48\pi\eta r_{sp}} \frac{d_p^2}{2dD} \tag{S8}$$

Thus, the capture rate in a 3-dimensional space ($d$ = 3) is:

$$k_{on} = 2\pi N_A R_{sp}^* D_{sp} c_{sp} = \frac{\alpha_1 QN_A d_p^2 E_0 c_{sp}}{144\eta r_{sp}} \tag{S9}$$

It clearly shows that the capture rate is proportional to the electric field inside the nanopore $E_0$ (*i.e.*, also to the applied voltage) and concentration of the DNA origami sphere $c_{sp}$.

## Supporting Note S2: Force balance of a docked DNA origami sphere

To estimate the support force given by the nanopore to a docked origami sphere, we consider the force balance of the origami sphere and the surrounding liquid electrolyte.

Let's first consider a simple electrophoresis scenario, an electrophoretically moving sphere in a channel filled with electrolyte (e.g. an aqueous salt solution), as shown in Fig. S1a. The sphere moves with a constant velocity with respect to the bulk electrolyte without net flow. Thus the electrophoretic force $F_{eph} = qE$ is equal to the shear force of the electrolyte $F_{s,sp} = 6\pi\eta v_0 r_{sp}$. The sphere velocity is $v_0 = qE/6\pi\eta r_{sp}$. If the channel wall is ideally smooth (full-slip wall condition), there is **no friction** between the electrolyte and the channel wall. Thus, the electrolyte can move freely with any velocity with respect to the channel wall. The forces on electrolyte are also balanced, *i.e.*, the electrical force on the counterions in the electrical double layer of the sphere surface $F_{ion}$ is equal to the friction force given by the moving sphere, $F_{fri,sp}$, considering the action-reaction force pairs, $F_{eph} = -F_{ion}$ and $F_{s,sp} = -F_{fri,sp}$. If, by some means, the sphere is stopped from moving and held statically with respect to that channel wall (Fig. S1b), the electrolyte will move with the same absolute velocity in the opposite direction, $-v_0$. In other words, **stopping an electrophoretically moving object will make the surrounding electrolyte move in the opposite direction, shifting from electrophoresis to electroosmosis**. After the sphere has been stopped and the electrolyte velocity has fully developed (stationary flow), the electrophoretic force on the sphere, $F_{eph}$, is balanced again by the shear force of the moving electrolyte, $F_{s,sp}$, with velocity $-v_0$, i.e., the electroosmotic flow (EOF) velocity. Thus, no extra force is needed to hold the sphere statically with respect to the channel wall.

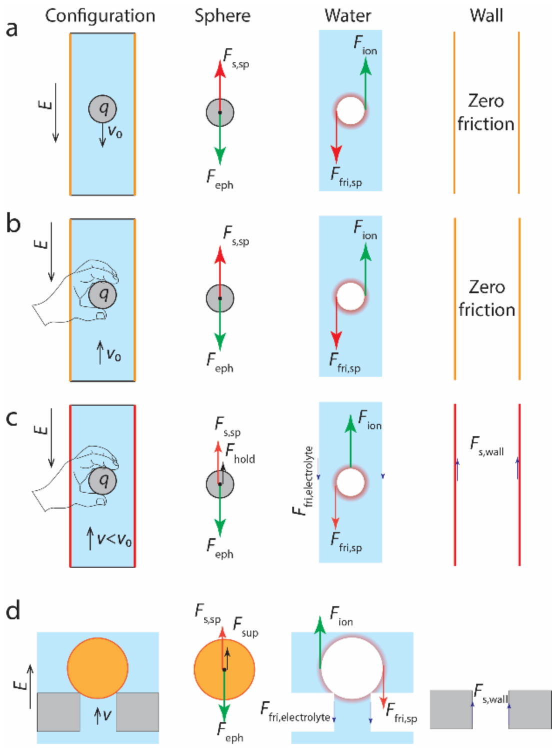


***Figure S1.*** *Schematics showing the force balance of electroosmotic systems. (a, b) A charged sphere in electrolyte in a the infinitely long tube without friction at the wall, in the condition of (a) the sphere moving and (b) the electrolyte moving, referring to the channel wall, respectively. (c) A charged sphere in a the infinitely long tube with non-slip wall. (d) A DNA origami sphere docked*

*on a nanopore. The first column shows the configurations of the discussed system, and the force balance on the sphere, electrolyte, and wall are showed in the second, third and last columns, respectively.*

However, if the channel wall is not ideally smooth (Fig. S1c), moving electrolyte generates frictional force to the wall, $F_{s,wall}$. The wall retards the moving electrolyte by the same force magnitude, $F_{fir,electrolyte}$ ($F_{s,wall}$ and $F_{fir,electrolyte}$ are an action-reaction force pair). Compared to the frictionless condition (Fig. S1b), the electrolyte velocity, $v$, will be smaller than $v_0$, and thus exert less shear force on the sphere. The consequence is that an extra force is needed to hold the sphere statically with respect to the channel wall. From the force balance on the sphere, this extra force to hold the sphere is the difference between the electrophoretic force and the shear force:

$$F_{hold} = F_{eph} - F_{s,sp} = qE - 6\pi\eta v r_{sp} = 6\pi\eta r_{sp}(v_0 - v) \quad \text{(S10)}$$

Considering the force balance on the electrolyte and action-reaction force pairs $F_{eph} = -F_{ion}$ and $F_{s,sp} = -F_{fri,sp}$, we have $F_{fri,electrolyte} = F_{fri,sp} - F_{ion} = -F_{s,sp} + F_{eph} = F_{hold}$. In other words, this holding force, $F_{hold}$, is exactly equal to the retardation force given by the channel wall towards the moving electrolyte, $F_{fri,electrolyte}$. Generally speaking, **the force to hold an object statically (with respect to the wall) in an electrolyte is equal to the retardation force given by the wall surface to the fluid which makes its EOF velocity, $v$, smaller than in the frictionless case, $v_0$.**

A similar picture can be applied to the NEOtrap system. As shown in Fig. S1d, for a docked DNA origami sphere, the support force provided by the nanopore to hold the sphere statically, $F_{sup}$, is exactly equal to the total retardation force from the fluidic wall (including surfaces of nanopore sidewall and the top/bottom surface of the membrane) to the moving electrolyte, $F_{fri,electrolyte}$. This retardation force is mainly contributed by the surface facing the fluidic with high flow rate, which is the nanopore sidewall in our NEOtrap system. This friction force from the sidewall acting on the electrolyte, $F_{fri,electrolyte}$, and the shear force given by EOF toward the sidewall, $F_{s,electrolyte}$, are an action-reaction force pair, and thus they are equal in amplitude.

In detail, the volume origami sphere can be divided into two parts, as shown in Fig. S2. The EOF from the center part (orange) in the projection area of the nanopore flows through the nanopore, and will be significantly slowed down by the nanopore sidewall (red surface) and thus contributes to the majority of the support force. The EOF from the peripheral part (blue) is distant from the surfaces (red and green surfaces), and thus we can assume that the EOF in this part is fully developed without significant retardation and does not significantly contribute to the support force. In our model, a factor, $V_r$, is introduce to describe the volumetric ratio of the central part (orange volume) to the total sphere (orange + blue volumes).

$$V_r = \frac{V_{center}}{V_{sp}} = \frac{\pi d_p^2 2r_{sp}/4}{4\pi r_{sp}^2/3} = \frac{3}{8}\left(\frac{d_p}{r_{sp}}\right)^2 \quad \text{(S11)}$$

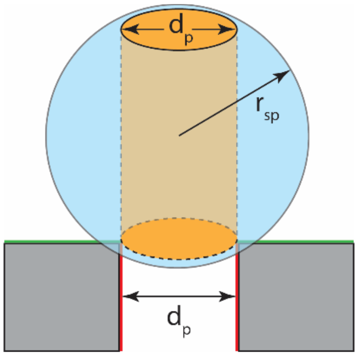


*Figure S2. Schematics showing the volume of a docked nanoporous DNA origami sphere projecting to the nanopore area (orange), in which the EOF is retarded significantly by the friction from the sidewall of nanopore (red area). thus the friction from sidewall contributes major support force. The blue volume is the peripheral part is distant from the surfaces (both red and green areas), and thus does not significantly cause friction. $d_p$ is the diameter of nanopore and $r_{sp}$ is the radius of the DNA-origami sphere.*

**Supporting Note S3: Electroosmotic flow calculation in NEOtrap system**

A pressure induced flow shows a parabolic velocity distribution over the cross-section of a cylindrical tube (Fig. S3a):

$$v_p(r) = v_{eof,p}\left(1 - \frac{4r^2}{d_p^2}\right) \tag{S12}$$

The fluid flux is:

$$Q_V = \int_0^{d_p/2} 2\pi r v_p(r) dr = \frac{\pi}{8} v_{eof,p} d_p^2 \tag{S13}$$

Thus, the average flow velocity is:

$$\bar{v}_p = \frac{4Q_V}{\pi d_p^2} = \frac{v_{eof.p}}{2} \tag{S14}$$

In addition, the shear stress on the surface is

$$\sigma_{para} = -\eta \left.\frac{dv_p(r)}{dr}\right|_{r=d_p/2} = \eta \frac{4v_{eof,p}}{d_p} \tag{S15}$$

Thus, the support force, which is equal to the shear force on the nanopore wall, is

$$F_{sup} = \pi d_p h \sigma_{para} = 4\pi h \eta v_{eof,p} \tag{S16}$$

For EOF in a cylindrical tube (Fig. S3b), the flow velocity profile is:[4]

$$v_c(r) = \left[1 - \frac{I_0(r/\lambda_D)}{I_0(r_{dna}/\lambda_D)}\right] v_{eof,c} \tag{S17}$$

where, $I_0$() is the modified Bessel function of order zero. Thus, the shear stress on the tubing wall is:

$$\sigma_{plug} = -\eta \frac{dv_c(r)}{dr}\bigg|_{r=r_{dna}} = \eta \frac{I_1(r_{dna}/\lambda_D)}{I_0(r_{dna}/\lambda_D)} \frac{v_{eof,c}}{\lambda_D} \tag{S18}$$

Thus, the shear force of the EOF to the DNA-origami sphere can be:

$$F_{eof} = 2\pi r_{dna} 2r_{sp} n\sigma_{plug} = 2\pi r_{dna} n 2r_{sp}\eta \frac{I_1(r_{dna}/\lambda_D)}{I_0(r_{dna}/\lambda_D)} \frac{v_{eof,c}}{\lambda_D} \approx 2\pi r_{dna} n 2r_{sp}\eta \frac{v_{eof,c}}{\lambda_D} \tag{S19}$$

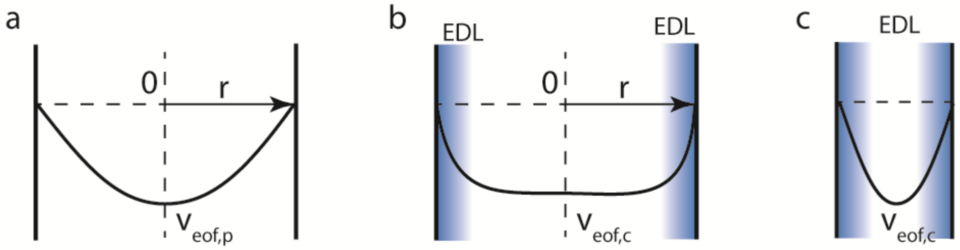


***Figure S3.*** *Schematics showing the velocity profiles in a cylindrical tube. (a) A pressure driven parabolic velocity distribution; (b) a plug flow velocity profile of EOF; (c) EOF in a very narrow tube where the EDL occupies most of the volume and the velocity profile are similar to a parabola.*

Considering the force balance of the DNA origami sphere, $F_{eph} = F_{eof} + F_{sup}$, we have

$$V_r \alpha_1 Q E_0 = 2\pi r_{dna} n 2r_{sp}\eta \frac{v_{eof,c}}{\lambda_D} + 4\pi h\eta v_{eof,p} \tag{S20}$$

The aqueous fluid volume is incompressible, and the flux through origami sphere is equal to that through the nanopore.

$$n\pi r_{dna}^2 \bar{v}_c = \pi d_p^2 \bar{v}_p / 4 \tag{S21}$$

Since in the nano-channels of the porous origami sphere, the electrical double layer (EDL) occupies almost all the volume (Fig. S3c), the average flow velocity can be approximated by the half of its maximum. Thus, we have:

$$n r_{dna}^2 v_{eof,c} = d_p^2 v_{eof,p} / 4 \tag{S22}$$

Combining Eq. S20 and Eq. S22, the EOF velocity inside de nanopore and nano-channel can be derived:

$$v_{eof,p} = -\frac{3}{8}\left(\frac{d_p}{r_{sp}}\right)^2 \frac{\alpha_1 Q E_0}{\pi\eta} \left(\frac{r_{sp} d_p^2}{r_{dna}\lambda_D} + 4h\right)^{-1} \tag{S23}$$

$$v_{eof,c} = -\frac{3}{8}\left(\frac{d_p}{r_{sp}}\right)^2 \frac{\alpha_1 Q E_0}{4\pi n\eta r_{dna}} \left(\frac{r_{sp}}{\lambda_D} + \frac{4hr_{dna}}{d_p^2}\right)^{-1} \tag{S24}$$

The ratio between the support force, $F_{sup}$, and the electrophoretic force, $F_{eph}$, is calculated, which represents the percentage of the driving force, $F_{eph}$, that is used for holding a docked sphere:

$$p = \frac{F_{sup}}{F_{eph}} = \frac{V_r}{1 + \frac{r_{sp} d_p^2}{4h\lambda_D r_{dna}}} \tag{S25}$$

**Supporting Note S4: Consideration of the slip wall condition**

For pressure induced flow in a nanopore with a parabolic velocity profile, the slip velocity is

$$u_{s,p} = -L_{s,p} \left.\frac{dv_p(r)}{dr}\right|_{r=d_p/2} = \frac{4L_{s,p}}{d_p} v_{eof,p0} \tag{S26}$$

where $L_{s,p}$ is the slip length of nanopore side wall. This slip velocity, $u_{s,p}$, playing as a background, superimposes on the EOF in the non-slip wall condition, $v_{eof,p0}$, by a systematic rise.

$$v_{eof,p} = v_{eof,p0} + u_{s,p} \tag{S27}$$

Let us reconsider the shear force generated by EOF through the nanopore which determines the support force:

$$F_{sup} = 4\pi h\eta \left(1 + \frac{4L_{s,p}}{d_p}\right) v_{eof,p} \tag{S28}$$

The EOF inside the nano-channels of the porous DNA origami sphere can be modeled by (Eq. S17) and the slip velocity is:

$$u_{s,c} = -L_{s,c} \left.\frac{dv_c(r)}{dr}\right|_{r=r_{dna}} = \frac{L_{s.c}}{\lambda_D}\left[\frac{I_1(r_{dna}/\lambda_D)}{I_0(r_{dna}/\lambda_D)}\right] v_{eof,c0} \tag{S29}$$

where $L_{s,c}$ is the slip length of nanochannel side wall. Similarly, we have

$$v_{eof,c} = v_{eof,c0} + u_{s,c} \tag{S30}$$

Thus, the shear force from the flow though the nano-channels in the origami sphere is:

$$F_{eof} = 4\pi n r_{dna} r_{sp} \eta \left[L_{s,b} + \frac{\lambda_D I_0(r_{dna}/\lambda_D)}{I_1(r_{dna}/\lambda_D)}\right]^{-1} v_{eof,b} \tag{S31}$$

Finally, considering the force balance on a docked DNA origami sphere:

$$V_r \alpha_1 QE = 4\pi n r_{dna} r_{sp} \eta \left[L_{s,b} + \frac{\lambda_D I_0(r_{dna}/\lambda_D)}{I_1(r_{dna}/\lambda_D)}\right]^{-1} v_{eof,b} + 4\pi h\eta \left(1 + \frac{4L_{s,p}}{d_p}\right) v_{eof,p} \tag{S32}$$

Combining Eq.S22 and Eq.S32 yields:

$$v_{eof,p} = \frac{V_r \alpha_1 Q E_0}{4\pi\eta} \left[ \frac{d_p^2 r_{sp}}{4r_{dna}\left( L_{s,b} + \frac{\lambda_D I_0(r_{dna}/\lambda_D)}{I_b(r_{dna}/\lambda_D)} \right)} + \frac{h}{1+\frac{4L_{s,p}}{d_p}} \right]^{-1} \quad \text{(S33)}$$

$$v_{eof,b} = \frac{V_r \alpha_1 Q E_0 d_p^2}{16 n\pi\eta r_{dna}} \left[ \frac{d_p^2 r_{sp}}{4r_{dna}\left( L_{s,b} + \frac{\lambda_D I_0(r_{dna}/\lambda_D)}{I_b(r_{dna}/\lambda_D)} \right)} + \frac{h}{1+\frac{4L_{s,p}}{d_p}} \right]^{-1} \quad \text{(S34)}$$

Thus, the ratio between the support force and the electrophoretic force is:

$$p = \frac{F_{sup}}{F_{eph}} = \frac{V_r}{1+\frac{r_{sp} d_p (d_p + 4L_{s,p})}{4hr_{dna}(\lambda_D I_0 / I_1 + L_{s,b})}} \quad \text{(S35)}$$

**Supporting Note S5. Distribution of electric field**

In a nanopore system, the electric field reaches its maximum inside the pore. It decays from the mouth of the pore to infinite distance. Considering the conservation of electric flux, the electric field intensity is reversely proportional to the area of equipotential surface. We can assume that the equipotential surface outside the nanopore access region is a hemisphere, where the electric field intensity will obey an inverse square relationship. It is worth noting that the docked negatively charge DNA origami sphere will only influence the local distribution of the electric field. In the long range, with the assistance of the counterion screening effect, the electric field intensity still decays in the inverse square relationship. In the access region ($0 \le z \le d_p/2$), a linearly decreasing field can be assumed. Thus, the distribution of the electric field can be expressed as follows:

$$E(z) = \begin{cases} E_0\left(1-\frac{z}{d_p}\right), (0 \le z < \frac{d_p}{2}) \\ E_0 \frac{d_p^2}{8z^2}, (\frac{d_p}{2} \le z < \infty) \end{cases} \quad \text{(S36)}$$

where, $E_0$ is the maximum electric field located at the pore mouth ($z$=0), which can be estimated by difference models[3]. For example, by consideration of the effective length of a nanopore $L_{eff}$, the maximum electric field $E_0$ under a bias voltage $V$ can be:

$$E_0 = \frac{V}{L_{eff}} \quad \text{(S37)}$$

And $L_{eff} = h + 0.92 d_p$ for a cylindrically shaped pore[5].

Similarly, all the conservative physical quantities, *e.g.*, water flow, that reach their peak value inside the pore can be approximated by an inverse square distribution. Thus, in the escape model, we assume a similar distribution of EOF velocity as the electric field here.

**Supporting Note S6: Charge of the dCas9-RNA-DNA complexes**

According to the sequence of the dCas9 from the UniProt (Q99ZW2), it carries ~20e positive charge at pH 7.5 condition calculated by the online tool Prot pi[1]. The length of crRNA and tracrRNA are 36 nucleotides (nt) and 67 nt, respectively. Thus, the dCas9-RNA complex carriers 20e-(36e+67e) = -83e charge, assuming one negative charge per phosphate group. Furthermore, for the dCas9-RNA-DNA complexes, the total charge number is –(83 + 2m)e, where m is the number of base pairs (bp) of the bound dsDNA. Thus, for the dCas9-RNA-DNA samples used in our experiments, the charges are -149e, -169e, -209e, and -283e, for dCas9-RNA-DNA33, dCas9-RNA-DNA43, dCas9-RNA-DNA63, and dCas9-RNA-DNA100, respectively.

**Supporting Note S7: Effective radius of the dCas9-RNA-DNA complexes**

According to the dCas9 structure from the Protein Data Bank (PDBID 6K57), it can be approximated as a ball-shape with 10 nm in diameter. If the length of the dsDNA is smaller than 10 nm, *i.e.*, 10 nm/0.33 nm/bp = 30 bp, it will not extend outside of the dCas9 and the complex keeps the shape of a 10 nm-diameter ball. Thus, in our model, the turning point between the ball model and the extended ball model appears at –(83+2×30)e = -143e, corresponding to the complex with a 30 bp dsDNA. In the extended ball model, the shape of the complex can be represented by a ball with an effective radius. The effective radius can be approximated by:

$$r_{a,eff} = 0.5\times(10\,[nm] + f_{eff}\,(m-30)\times 0.33\,[nm]) \quad \text{(S38)}$$

where, m is the length of the dsDNA in number of bp and $f_{\text{eff}}$ is a factor converting real length to effective radius. The unit of this formula is [nm] and $f_{\text{eff}}$ is a free parameter during fittings.

[1] https://www.protpi.ch/Calculator/ProteinTool

## Supporting Figures

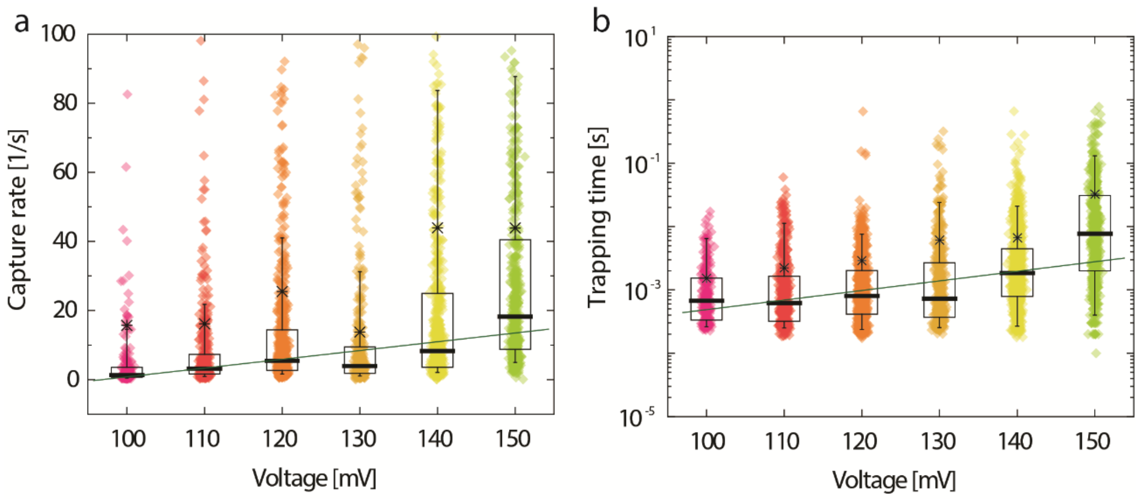


**Figure S4.** Trapping protein Ovalbumin at various voltages. (a) Capture rate and (b) trapping time of Ovalbumin trapped in a 10 nm-diameter nanopore. The concentration of Ovalbumin is 23 nM. Each dot represents an individual trapping event and the box chart shows the mean (asterisk), median (thick bar), and the 10%, 25%, 75%, and 90% levels. The solid line in (a) shows a linear fitting result, while the solid line in (b) shows an exponential fitting result, of the median values in both cases.

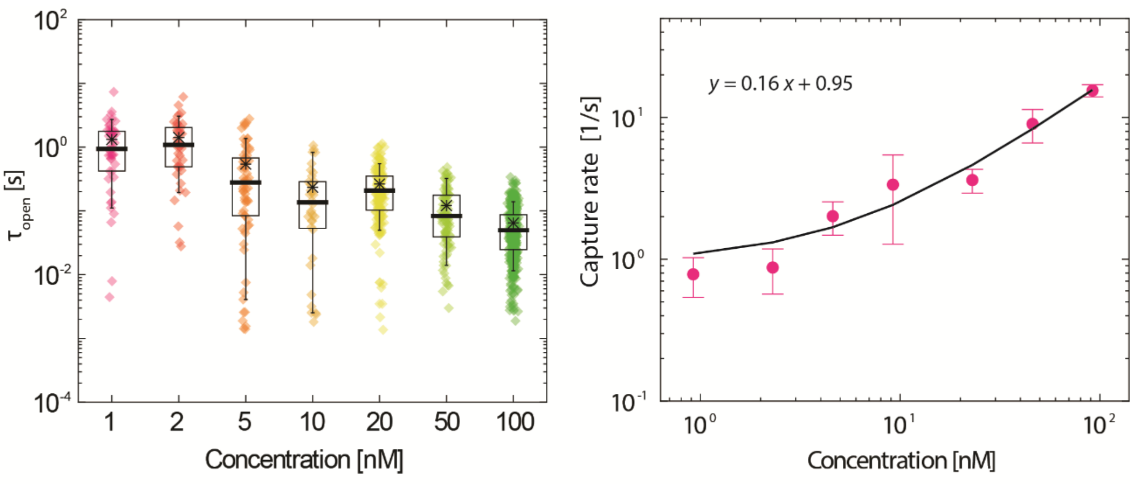


**Figure S5.** Trapping of different concentrations of Ovalbumin with a 10 nm-diameter nanopore at 100 mV bias voltage. (a) Waiting time of trapping (*i.e.*, the time that no protein in the pore). Each dot represents an individual trapping event and the box chart shows the mean (asterisk), median (thick bar), and the 10%, 25%, 75%, and 90% levels. (b) The capture rate as a function of protein concentration. Bootstrap sampling was used for the data in (a) to get the mean and standard deviation values: for a set of M data points, 10 subsets with a size of 60% of M were randomly picked, fitted by an exponential distribution separately. Then, the means and standard deviations across these subsets are calculated. The solid line shows a linear fit using the indicated equation.

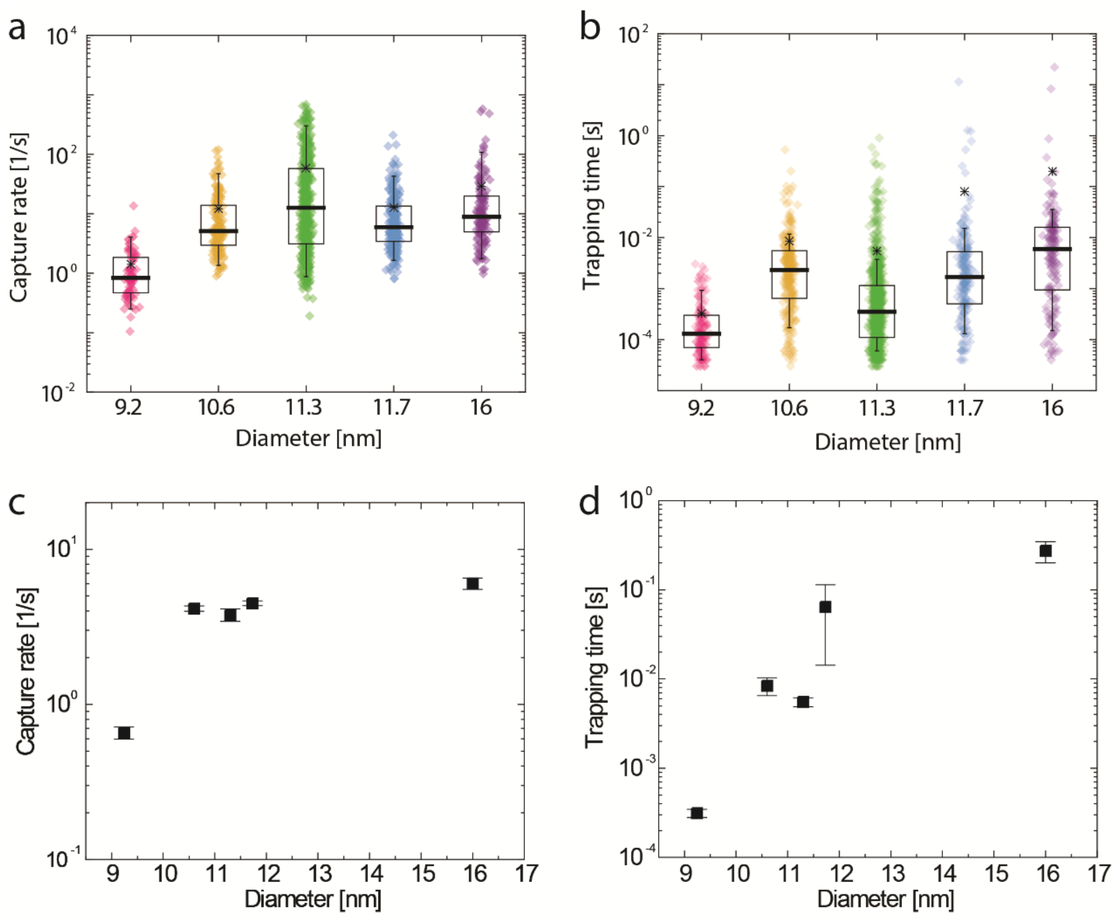


**Figure S6.** Nanopore diameter dependence of Ovalbumin trapping. (a, b) Capture rate and trapping time for 23 nM Ovalbumin at 100 mV by nanopores with different diameters, respectively. Each dot represents an individual trapping event and the box chart shows the mean (asterisk), median (thick bar), and the 10%, 25%, 75%, and 90% levels. (c, d) The capture rate and trapping time are plotted as a function of nanopore diameter. The capture rate and trapping time are extracted from exponential fitting of the waiting time distributions (*i.e.*, the reciprocal of capture rate in (a) and trapping time data (b), respectively). In order to estimate the uncertainties, bootstrap sampling was used: for a set of M data points, 10 subsets with a size of 60% of M were randomly picked, fitted by an exponential distribution separately. Then, the means and standard deviations across these subsets are calculated.

References:


(1) Wen, C.; Bertosin, E.; Shi, X.; Dekker, C.; Schmid, S. Orientation-Locked DNA Origami for Stable Trapping of Small Proteins in the Nanopore Electro-Osmotic Trap. *Nano Lett.* **2023**, *23* (3), 788–794. https://doi.org/10.1021/acs.nanolett.2c03569.
(2) Schmid, S.; Stömmer, P.; Dietz, H.; Dekker, C. Nanopore Electro-Osmotic Trap for the Label-Free Study of Single Proteins and Their Conformations. *Nat. Nanotechnol.* **2021**, *16* (11), 1244–1250. https://doi.org/10.1038/s41565-021-00958-5.
(3) Wen, C.; Zhang, S.-L. Fundamentals and Potentials of Solid-State Nanopores: A Review. *J. Phys. D: Appl. Phys.* **2021**, *54* (2), 023001. https://doi.org/10.1088/1361-6463/ababce.
(4) Bruus, H. Chapter 9. Electroosmosis. In *Theoretical Microfluidics*; Oxford University Press: New York, 2008; pp 157–170.
(5) Wen, C.; Zhang, Z.; Zhang, S.-L. Physical Model for Rapid and Accurate Determination of Nanopore Size via Conductance Measurement. *ACS Sens.* **2017**, *2* (10), 1523–1530. https://doi.org/10.1021/acssensors.7b00576.
